# Evolutionary Responses to a Constructed Niche: Ancient Mesoamericans as a Model of Gene-Culture Coevolution

**DOI:** 10.1371/journal.pone.0038862

**Published:** 2012-06-21

**Authors:** Tábita Hünemeier, Carlos Eduardo Guerra Amorim, Soledad Azevedo, Veronica Contini, Víctor Acuña-Alonzo, Francisco Rothhammer, Jean-Michel Dugoujon, Stephane Mazières, Ramiro Barrantes, María Teresa Villarreal-Molina, Vanessa Rodrigues Paixão-Côrtes, Francisco M. Salzano, Samuel Canizales-Quinteros, Andres Ruiz-Linares, Maria Cátira Bortolini

**Affiliations:** 1 Departamento de Genética, Instituto de Biociências, Universidade Federal do Rio Grande do Sul, Porto Alegre, Rio Grande do Sul, Brazil; 2 Centro Nacional Patagónico, CONICET, U9120ACV, Puerto Madryn, Argentina; 3 Molecular Genetics Laboratory, Escuela Nacional de Antropología e Historia, Mexico City, Mexico; 4 Programa de Genética Humana, Instituto de Ciencias Biomédicas, Facultad de Medicina, Universidad de Chile, Santiago, Chile; 5 Instituto de Alta Investigación, Universidad de Tarapacá, Arica, Chile; 6 Laboratoire d′Anthropologie Moléculaire et d′Imagerie de Synthèse, UMR 5288 CNRS, Université Paul Sabatier (Toulouse3), Toulouse, France; 7 Anthropologie Bio-culturelle, Droit, Ethique et Santé (ADES), UMR 7268, Aix-Marseille-Université/CNRS/EFS, Marseille, France; 8 Escuela de Biología, Universidad de Costa Rica, San José, Costa Rica; 9 Laboratorio de Genómica de Enfermedades Cardiovasculares, Instituto Nacional de Medicina Genómica, Mexico City, Mexico; 10 Unit of Molecular Biology and Genomic Medicine, Instituto Nacional de Ciencias Médicas y Nutrición Salvador Zubirán, Mexico City, Mexico; 11 Departamento de Biología, Facultad de Química, Universidad Nacional Autónoma de México, Mexico City, Mexico; 12 The Galton Laboratory, Department of Biology, University College London, London, United Kingdom; University of Cambridge, United Kingdom

## Abstract

Culture and genetics rely on two distinct but not isolated transmission systems. Cultural processes may change the human selective environment and thereby affect which individuals survive and reproduce. Here, we evaluated whether the modes of subsistence in Native American populations and the frequencies of the ABCA1*Arg230Cys polymorphism were correlated. Further, we examined whether the evolutionary consequences of the agriculturally constructed niche in Mesoamerica could be considered as a gene-culture coevolution model. For this purpose, we genotyped 229 individuals affiliated with 19 Native American populations and added data for 41 other Native American groups (n = 1905) to the analysis. In combination with the SNP cluster of a neutral region, this dataset was then used to unravel the scenario involved in *230Cys* evolutionary history. The estimated age of *230Cys* is compatible with its origin occurring in the American continent. The correlation of its frequencies with the archeological data on *Zea* pollen in Mesoamerica/Central America, the neutral coalescent simulations, and the F_ST_-based natural selection analysis suggest that maize domestication was the driving force in the increase in the frequencies of *230Cys* in this region. These results may represent the first example of a gene-culture coevolution involving an autochthonous American allele.

## Introduction

Human cultural practices have drastically modified environmental conditions and behaviors, promoting rapid and substantial genomic changes often associated with positive selection and adaptation (gene-culture dynamics [1], [2]). In the history of *Homo sapiens sapiens*, a particularly important event that triggered a new and striking gene-culture-coevolution cycle was the development of agriculture and animal domestication during the Neolithic period (∼10,000 years ago). Further, the human gene-culture coevolution mediated by the domestication of plants and animals has been argued to provide some of the clearest and most spectacular examples of niche construction. The Niche Construction Theory can be defined as a branch of evolutionary biology that emphasizes on the ability of organisms to modify the pressure of natural selection in their environment and thereby act as co-directors of their own evolution, as well as that of other directly associated species [3]–[6].

Although more than 100 regions/genes had been identified as the likely targets of recent positive selection resulting from cultural pressures in newly constructed niches [1], well-documented examples are scarce. One of the best-known cases of gene-culture coevolution is lactase persistence (LP; the ability of adult humans to digest the lactose found in fresh milk) and dairying. High frequencies of LP are generally observed in traditional pastoralist populations. For example, LP reaches ∼64% in Beni Amir pastoralists from Sudan, whereas its frequency in a neighboring non-pastoralist community is only ∼20%. In Europe, LP varies from 15–54% in eastern and southern regions, 62–86% in central and western regions, and 89–96% in northern regions [5], [7]–[13]. Multiple independent mutations have been associated with this characteristic, some of which are located in an intron of the *MCM6* gene, a region fundamental to lactase expression [5], [14]. The alleles that led to lactose persistence in Europe, such as MCM6 13,910*T, first underwent selection among dairying farmers around 7,500 years ago, possibly in association with the dissemination of the Neolithic Linearbandkeramik culture over Central Europe [13]. The high copy number variation of the amylase gene and the spread of the corresponding alleles in agricultural societies are another well-studied example [7], [8], [11], [15], [16]. Additionally, the West African Kwa-speaking agriculturalists cut and clear the forest to grow yams, increasing the amount of standing water after rain, therefore providing better breeding grounds for malaria-carrying mosquitoes [1] favoring the *HbS* allele, which confers protection against malaria in heterozygous individuals [17].

America was the last continent colonized by modern humans in prehistoric times. In less than 15,000 years before present (YBP), these first migrants had to adapt to an immensely wide variety of environments. In some regions during this evolutionary trajectory, as in Mesoamerica and the Andes, hunter-gatherer/forager societies gave rise to agriculturalist and urban communities, while others remained with a hunter-gatherer/forager subsistence system until the time of contact with Europeans or even until the present day. Thus, studies with Native American populations can provide useful information for better understanding gene-culture coevolution and the niche construction processes.

Based on studies with blood groups and other classical genetic polymorphisms, J. V. Neel and F. M. Salzano were pioneers in identifying complex population processes highly dependent on cultural factors in Native Americans (e.g., fission-fusion dynamic; [18]). Other examples are related to the coevolution of genes and languages [19], [20], but only two more recently reported examples might be associated with positive selection: (1) Tovo-Rodrigues *et al.* [21] investigated the distribution of D4 dopamine receptor (*DRD4*) alleles in several South Amerindian populations and found a significant difference in the allelic distributions between hunter-gatherers and agriculturalists, with an increase of the *7R* allele among the former; and (2) Acuña-Alonzo *et al*. [22] showed that the *230Cy*s allele (*Arg230Cys*, rs9282541) of the ATP-binding cassette transporter A1 (*ABCA1*) gene, which was previously associated with low HDL-cholesterol levels and obesity-related comorbidities, was exclusively present in Native American and mestizo individuals. These authors verified that cells expressing the ABCA1*230Cys allele showed a 27% cholesterol efflux reduction, confirming that this Native American autochthonous variant has a functional effect *in vitro*. Other investigations have shown that the presence of ABCA1*230Cys explains almost 4% of the variation in plasma HDL-C concentrations in Mexican admixed populations [23]. This variation in HDL-C concentration was the highest one associated with a single nucleotide polymorphism (SNP) among different continental populations in these genome-wide association studies, corroborating its functionality [23].

Acuña-Alonzo *et al.* [22] demonstrated that *230Cys* resides in a haplotype that was the target of an ongoing directional selective sweep, suggesting that *230Cys* conferred an advantage during periods of food deprivation in the past. On the other hand, under the current modern lifestyle, *230Cys* may have become a major susceptibility allele for low HDL levels and has been correlated with metabolic diseases [22]. This study provides an example of the “thrifty” genotype hypothesis, which postulates that variants that increase the efficiency of energy use and storage during periods of famine would have been positively selected in prehistoric times but can be associated with diseases of affluence in contemporary societies, where food is usually abundant [24].

Here, we expand the investigations of the *Arg230Cys* polymorphism in Native Americans and integrate the thrifty genotype concept with the gene-culture coevolution process, considering the human ability to create new ecological niches that may lead to the selection of genetic variants.

## Materials and Methods

### (a) Populations

New data for the *Arg230Cys* polymorphism were generated for 19 Amerindian populations (n = 229) from Meso/Central America and South America (Table 1). Additional information about these tribes can be found in Bortolini *et al.* [25], [26], Wang *et al*. [27], and Mazières *et al.* [28], [29]. These new *Arg230Cys* data were then analyzed together with those of an earlier published report [22], providing a total of 1905 investigated individuals. One hundred and twenty-six individuals of our sample were also previously genotyped for ∼680,000 SNPs using Illumina Human 610-Quad BeadChips(Ruiz-Linares *et al.*, unpublished data), and a part of this information was used in some of our analyses. The populations were clustered according to their geographical location and ancient mode of subsistence as follows: (1) Mesoamerican agriculturalists, (2) Andean agriculturalists, and (3) South American hunter-gatherers/foragers. Of course, caution is needed regarding this classification, since subsistence modes are not stable over time and may not be unique. However, the two categories adopted here (agriculturalists and hunter-gatherers/foragers) represent general pre-Colombian subsistence conditions, providing a starting point for research related to gene-culture dynamics in Native Americans.

**Table 1:** Genotypes, allele frequencies, and geographic locations of the Native American populations investigated.

Population	*N* 1	Genotype frequency				Allele frequency		Country	Geographicalcoordinates	References
		*Arg* *230* *Arg*	*Arg* *230* *Cys*	*Cys* *230* *Cys*	*Arg230*		*230Cys*			
**Mesoamerican agriculturalist** 2 **(1218)**										
Yaqui	45	30	11	4	0.79		0.21	Mexico	27° 29′ N 110° 40′ W	Acuña-Alonzo et al. (2010)
Tarahumara	109	81	23	5	0.85		0.15	Mexico	26° 49′ N 107° 04′ W	Acuña-Alonzo et al. (2010)
Teenek	67	45	20	2	0.82		0.18	Mexico	21° 36′ N 98° 58′ W	Acuña-Alonzo et al. (2010)
Cora	123	62	51	10	0.71		0.29	Mexico	22° 3′ N 104° 55′ W	Acuña-Alonzo et al. (2010)
Purepecha	35	22	11	2	0.79		0.21	Mexico	19° 36′ N 102° 14′ W	Acuña-Alonzo et al. (2010)
Mazahua	83	68	15	0	0.91		0.09	Mexico	19° 26′ N 100° 00′ W	Acuña-Alonzo et al. (2010)
Mixe	19	15	4	0	0.89		0.11	Mexico	17° N 96°W	Present study
Mixtec	4	4	0	0	1.00		0.00	Mexico	17° N 97°W	Present study
Nahuatl	267	185	73	9	0.83		0.17	Mexico	19° 58′ N 97° 37′ W	Acuña-Alonzo et al. (2010)
Totonaco	113	86	24	3	0.87		0.13	Mexico	19° 57′ N 97° 44′ W	Acuña-Alonzo et al. (2010)
Otomíes	42	35	7	0	0.92		0.08	Mexico	20° 28′ N 99° 13′ W	Acuña-Alonzo et al. (2010)
Zapotec	125	71	50	4	0.76		0.24	Mexico	17°14′ N 96°14′ W	Present study; Acuña-Alonzo et al. (2010)
Mayan	110	68	39	3	0.80		0.20	Mexico	20°13′ N 90°28′ W	Acuña-Alonzo et al. (2010)
Kaqchikel-Quiche	17	13	3	1	0.85		0.15	Guatemala	15° N 91°W	Present study
Cabecar	24	19	5	0	0.90		0.10	Costa Rica	9° 30′ N 84°W	Present study
Guaymí	35	26	8	1	0.85		0.15	Costa Rica/Panamá	8°30′ N 82° W	Present study
**South American hunter-gatherer/forager** 2 **(572)**										
Parkatejê (Gavião)	78	65	12	1	0.91		0.09	Brazil	05° 03′ S 48° 36′ W	Acuña-Alonzo et al. (2010)
Jamamadi	26	26	0	0	1.00		0.00	Brazil	07° 15′ S 66° 41′ W	Acuña-Alonzo et al. (2010)
Mekranoti (Kayapó)	25	24	1	0	0.98		0.02	Brazil	08° 40′ S 54° W	Acuña-Alonzo et al. (2010)
Mura (Pirahã)	18	11	6	1	0.78		0.22	Brazil	03°34′ S 59° 12′ W	Acuña-Alonzo et al. (2010)
Pacaás-Novos (Wari)	25	23	2	0	0.96		0.04	Brazil	11° 08′ S 65° 05′ W	Acuña-Alonzo et al. (2010)
Sateré-Mawé	25	20	4	1	0.88		0.12	Brazil	03° S 57° W	Acuña-Alonzo et al. (2010)
Apalaí	22	15	7	0	0.84		0.16	Brazil	01°20′ N 54°40′ W	Acuña-Alonzo et al. (2010)
Arara	24	15	9	0	0.81		0.19	Brazil	03° 30′ S 54°10′ W	Acuña-Alonzo et al. (2010)
Guarani	31	30	1	0	0.98		0.02	Brazil	25° 20′ S 52° 30′ W	Present study; Acuña-Alonzo et al. (2010)
Gorotire (Kayapo)	7	6	0	1	0.86		0.14	Brazil	07° 44′ S 51° 10′ W	Acuña-Alonzo et al. (2010)
Karitiana	20	20	0	0	1.00		0.00	Brazil	08° 45′ S 63° 51′ W	Acuña-Alonzo et al. (2010)
Xavante	21	10	9	2	0.69		0.31	Brazil	13° 20′ S 51° 40′ W	Acuña-Alonzo et al. (2010)
Xikrin (Kayapo)	17	16	1	0	0.97		0.03	Brazil	05°55′ S 51°11′ W	Acuña-Alonzo et al. (2010)
Yanomama	25	20	4	1	0.88		0.12	Brazil	02°30 ′–04°30′ N 64° W	Acuña-Alonzo et al. (2010)
Txukahamae (Kayapo)	30	26	4	0	0.93		0.07	Brazil	10° 20′ S 53° 5′ W	Acuña-Alonzo et al. (2010)
Tiriyó (Trio)	25	21	4	0	0.92		0.08	Brazil	01° 57′ N 55°49′ W	Acuña-Alonzo et al. (2010)
Içana River (Baniwa)	19	13	3	3	0.76		0.24	Brazil	01° N 67° 50′ W	Acuña-Alonzo et al. (2010)
Kuben Kran Keng(Kayapo)	17	13	4	0	0.88		0.12	Brazil	08°10′ S 58°8′ W	Acuña-Alonzo et al. (2010)
Lengua	29	29	0	0	1.00		0.00	Paraguay	23° S 56° W	Acuña-Alonzo et al. (2010)
Ache (Guayaki)	23	23	0	0	1.00		0.00	Paraguay	23° S 58° W	Acuña-Alonzo et al. (2010)
Ayoreo	30	30	0	0	1.00		0.00	Paraguay	16–22° S 58–63° W	Acuña-Alonzo et al. (2010)
Zenu	4	4	0	0	1.00		0.00	Colombia	9° N 75° W	Present study
Kogi	7	7	0	0	1.00		0.00	Colombia	11° N 74° W	Present study
Ticuna	1	1	0	0	1.00		0.00	Colombia	3° 53′ S 70°W	Present study
Embera	3	3	0	0	1.00		0.00	Colombia	7° N 76° W	Present study
Wayuu	17	15	2	0	0.94		0.06	Colombia	11° N 73° W	Present study
Palikur	3	1	2	0	0.67		0.33	French Guiana	4° N 51° 45′ W	Present study
**Andean agriculturalist** 2 **(115)**										
Mapuche	40	40	0	0	1.00		0.00	Chile	40° 30′ S 69° 20′ W	Acuña-Alonzo et al. (2010)
Aymara	16	16	0	0	1.00		0.00	Bolivia	16°30′ S 68°9′ W	Present study
Quechua	16	15	1	0	0.97		0.03	Bolivia	14°30′ S 69° W	Present study
Aymara	22	20	2	0	0.95		0.05	Chile	22° S 70° W	Present study
Chilote	2	2	0	0	1.00		0.00	Chile	42°30′ S 73°55′ W	Present study
Hulliche	13	10	3	0	0.89		0.11	Chile	41° S 73° W	Present study
Ingano	6	5	1	0	0.92		0.08	Colombia	1° N 77° W	Present study

1 Samples genotyped in present study  = 229;

2 Caution is needed regarding the classification of these modes of subsistence, since they are not stable over time and may not be unique. However, the two categories adopted here (agriculturalist and hunter-gatherer/forager) represent general pre-Columbian subsistence conditions of the investigated populations in accordance with what is known about them. AMOVA results: (a) Among the subdivisions (*F_CT_*): 3.6% (*p* = 0.000); (b) Among populations within the Mesoamerican Agriculturalist subdivision (*F_ST_):* 1.8% (*p* = 0.008); (c) Among populations within the South American hunter-gatherer/forager subdivision: 5.3% (*p* = 0.005); Among populations within the Andean Agriculturarist group: 0% (*p* = 0.36).

Ethical approval for the present study was provided by the Brazilian National Ethics Commission (CONEP Resolution no. 123/98) for the Brazilian samples, as well as by ethics committee of: (a) Universidad de Antioquia, Medellin, Colombia (Colombian samples); (b) Universidad Nacional Autónoma de México, Ciudad de México, México (Mexican samples); (c) Universidad de Costa Rica, San José, Costa Rica (Costa Rican and Panamanian samples); (d) Universidad of Chile, Santiago, Chile (Chilean samples); (e) Université Paul Sabatier Toulouse 3, Toulouse, France (Bolivian and French Guianian samples). Individual and tribal informed oral consent was obtained from all participants, since they were illiterate, and they were obtained according to the Helsinki Declaration. The ethics committees approved the oral consent procedure as well as the use of these samples in population and evolutionary studies.

### (b) SNP Genotyping and Intra- and Inter-subdivision Structures

The *Arg230Cys* polymorphism was genotyped using TaqMan assays (ABI Prism 7900HT Sequence Detection System; Applied Biosystems). Allele frequencies were obtained by direct counting. The level of the population structure observed within and between Mesoamerican agriculturalist, Andean agriculturalist, and South American hunter-gatherer/forager groups was estimated using *F* statistics and the Arlequin 3.5.1 software [30]. Allele frequencies were compared between the 3 population subdivisions with the student’s *t*-test (alfa  = 0.05) using the R Stats package (R Development Core Team; http://www.R-project.org/).

### (c) Allele Age and Neutrality/selection Tests

A large Asian and Native American sample, including the 126 individuals investigated here, were genotyped for a major panel of ∼680,000 SNPs (Ruiz-Linares *et al*., unpublished data). Based on this additional information (Tables S1, S2 and S3), the following analyses were performed:

#### (c.1) ABCA1*230Cys allele age

Since estimates of allele age depend on assumptions about demographic history and natural selection, we have performed two approaches to estimate the age of the variant allele:

1. Kimura and Ohta [31] were the first to consider the relations between allele age and its frequency. With this purpose they developed the equation *E*(*t*1)  =  [−2*p*/(1−*p*)]ln(*p*), where *E*(*t*1)  =  expected age, time is measured in units of 2*N* generations, and *p*  =  population frequency [31]. For ABCA1*230Cys, we considered the average of frequencies of all populations and only those from Mesoamerica/Central America (*p* = 9.6 and 15.4, respectively; Table 1). A generation time of 25 years and *N* = 720 (number of generation considering the upper limit for the peopling of America, 18,000 YBP [32]) were assumed.
2. Slatkin and Rannala [33] began to exploit Linkage Disequilibrium (LD) to estimate allele ages, based on variation among different copies of the same allele, where the age of an allele is estimated by the intra-allelic variation following the LD exponential decay due to recombination and mutation rates. Rannala and Reeve [34], on the other hand, explored the use of LD to map genes, as well as to obtain the allele age using a Markov Chain Monte Carlo framework. We applied this method to obtain a second ABCA1*230Cys age estimative using the DMLE+ v2.2 software (http://www.dmle.org). This program allows a Bayesian inference of the mutation age using an intra-allelic coalescent model to assess LD across the nineteen SNPs that occur around ABCA1*230 (rs2065412, rs2515601, rs2472386, rs2274873, rs2487054, rs4149290, rs2487039, rs2472384, rs2253174, rs2230806, rs2230805, rs2249891, rs4149281, rs4743764, rs1929841, rs2000069, rs2275542, rs3904998, and rs4149268). Taken into account the historical information about our sampled populations, three parameters were introduced: (a) Generation time of 25 years; (b) Proportion of population growth of 0.005; and (c) Proportion of population sampled of 0.0002. The program was run in the haplotype mode using two million of iterations.

#### (c.2) Test to detect deviations from neutrality

Based on the long-range haplotype test [35] and integrated haplotype scores [9], [36] Acuña-Alonzo et al. [22] suggested that the autochthonous Native American ABCA1*230Cys allele could have been positively selected. However, demographic events, population structure, and other stochastic processes can create complex patterns in the genome, obscuring signals of natural selection or mimicking adaptive processes [37]. Additionally, positive and balancing selections show different effects on the genetic diversity patterns within and between populations [38]. Therefore, we performed additional analyses to explore these issues and elucidate the factors responsible for the eventual effect of natural selection on the ABCA1*230 locus.

To detect loci under selection, we used a method that contrasted the observed population differentiation (*F_ST_*) with that generated for a null simulated distribution under a hierarchical island model using a coalescent approach. In this model, demes exchange more migrants within groups than between groups to generate the joint distribution of genetic diversity within and between populations [38]. Thus, a *p* value can be estimated from the joint distribution for the population heterozygosity (*H_e_*) and *F_ST_* using a kernel density estimation procedure [30]. The analysis was performed using Arlequin 3.5.1 in consideration of 126 Native Americans whose results for the ABCA1*230 locus were known. Data from 20 other autosomal SNPs (rs6559725, rs11140096, rs4877767, rs4014024, rs11140109, rs7872891, rs7850633, rs17086298, rs10746709, rs5014093, rs10868019, rs11140116, rs3860938, rs3860941, rs4097644, rs9942844, rs12551103, rs7863524, rs4877785, and rs70439590) from these same individuals were compiled from a major SNP panel (Table S2). These 20 additional SNPs were selected based on their location (chromosome 9: from position 85252250 to 85317359) inside the putative neutral region, defined by Schroeder and colleagues [39], which comprises ∼76,000 bp around the *D9S1120* locus. Using this database (Table S2 and Figure S1), we were able to evaluate whether the joint distribution of the observed *H_e_* and *F_ST_* for the ABCA1*230 polymorphism data departed from the expected outcome at neutrality. Fifty thousand coalescent simulations were performed with a 100-demes island model. Four comparative analyses were conducted: (1) Mesoamerican agriculturalists (n = 68) *vs.* Andean agriculturalists (n = 35), (2) Mesoamerican agriculturalists *vs.* South American hunter-gatherers/foragers (n = 23), (3) Mesoamerican agriculturalists *vs.* South Americans (agriculturalists + hunter-gatherers/foragers), and (4) Andean agriculturalists *vs.* South American hunter-gatherer/foragers.

In addition, we simulated 100,000 neutral genealogies for a region containing two distinct sets of 20 biallelic markers under demographic scenarios mimicking the settlement of the Americas [32], [40]. To accomplish these simulations, we used the msABC software [41], a modification of ms software [42] that uses the coalescent to generate samples under a neutral Wright-Fisher model. The demographic parameters included: (1) a current effective population size of 830 individuals [40], a number not very different from that used in the simulations of Schroeder *et al.* [39]; (2) three demes that corresponded to the sampled subdivisions (Mesoamerican agriculturalists, Andean agriculturalists, South American hunter-gatherers/foragers); (3) a single ancestral population that existed from 6,350 to 18,000 YBP [32], [40]; and (4) different exponential growth rates to include the possibility of an ancestral population of 70 to 830 individuals (i.e., constant population size [40]).

The genetic diversity obtained in the simulations–summarized by intra- (heterozygosity) and interpopulation (global and pairwise *F_ST_*) statistics–was compared to the observed genetic diversity of two genetic datasets: (1) that of the ABCA1*230 locus plus the same 19 flanking SNPs listed in item c.1; (Table S3), and (2) the same 20 SNPs mentioned in section c.2 (Table S2; Figure S1). The summary statistics calculated for each simulation (S vectors) were then compared to the summary statistics of the observed data (S* vectors) using an Euclidean distance measure ∂  =  ||S-S*|| with the ABCestimator software, implemented with the ABCtoolbox [43]. The rationale of the analysis was to check which observed dataset could be reproduced with higher fidelity among the range of neutral simulations.

### (d) Allele Frequencies vs. Maize Domestication

Genetic, archeological, botanical, and paleoecological data furnished evidence that maize (*Zea mays ssp. mays*) had a single domestication origin from the wild grass teosinte (*Zea mays ssp. parviglumis*) in the Río Balsas region, southwestern Mexico, approximately 6,300–10,000 calendar years before present [44]–[53]. Pollen samples taken from sediments in lakes, swamps, and archeological deposits have provided evidence for the presence or absence of *Zea* (maize and/or teosinte) in the Americas and have been used to estimate the age of maize domestication and dispersion [44]. Blake [44] summarized the *Zea* pollen dates from several American archeological sites, and we selected this data set to perform our analysis. To test the connection between maize culture and the ABCA1*230Cys variant, we used allele frequencies from Mesoamerica/Central America populations (Zapotec, Maya, Nahuatl,Kaqchikel-Quiche, Totonac, Cabecar, and Guaymí) as well as *Zea* pollen dates obtained in archeological sites located geographically near these populations (Table 2). Spearman rank order correlations between the two data sets (*Zea* pollen archeological records and ABCA1*230Cys allele frequencies) were obtained using the Statistica 7.0 software (StatSoft, Inc©).

**Table 2:** Zea pollen relics ages and 230Cys*ABCA1 populations frequencies used for the regression analysis.

Native Americans		Zea Pollen relics		Site Ages (BP)^2^		Allele frequency
Population^1^	Geographic region	Archeological site	Geographic region	Radiocarbon years	Calendar years	230Cys*ABCA1
Zapotec	Oaxaca	Guilá Naquitz	Oaxaca	8240	9212	0.24
Maya	Tabasco	San Andrés	Tabasco	6208	7122	0.20
Nahuatl	Mexico state	Zoalpilco	Mexico state	5090	5835	0.17
Kaqchikel-Quiche	Guatemala	Zipacate	Guatemala	4600	5318	0.15
Totonac	Veracruz	Laguna Pompal	Veracruz	4250	4818	0.13
Cabecar	Costa Rica	Lago Cote	Costa Rica	2940	3096	0.10
Guaymí	Panama/Costa Rica	Gatun Lake	Panamá	4000	4468	0.15

1 Located near the archeological sites of *Zea* pollen relics; 2 Conversion according to www.radiocarbon.ldeo.columbia.edu/radcarbcal.htm.

2 Data relative to archeological information were obtained from Blake (2006).

## Results

### SNP Genotyping and Intra- and Inter-group Structures

Table 1 presents the genotype and allele frequencies for the 1905 individuals analyzed, including the new samples genotyped in the present study. A molecular analysis of variance (AMOVA) test was performed to quantify the level of population structure observed within and between the 3 subdivisions adopted here (Mesoamerican agriculturalists, Andean agriculturalists, and South American hunter-gatherer/forager; Table 1). A significant difference was observed between the subdivisions (*F_CT_*  = 0.036; *p* = 0.000). On the other hand, the highest *F_ST_* among populations within subdivisions was observed in the South American hunter-gatherers/foragers (0.053; *p = *0.005), and a value 5 times lower was found among Mesoamerican agriculturalists (0.013; *p* = 0.008); no sign of structuration was found in the Andes area (*p* = 0.36).

No significant differences in allelic frequencies were found when subsistence modes (hunter-gatherer/foragers *vs.* agriculturalists) were compared using the student’s *t*-test (*p* = 0.1316; Table 1). However, significant differences were observed between Mesoamerican agriculturalists and Andean agriculturalists or South American hunter-gatherers/foragers (*p* = 0.0022 and *p* = 0.0174, respectively). A comparison of South American hunter-gatherers/foragers and Andean agriculturalists revealed no significant differences (*p* = 0.351).

#### (c.1) ABCA1*230Cys allele age

The ABCA1*230Cys allele age estimates, using population frequency information, were 12,097 YBP and 19,409 YBP, considering data from all populations and only those from Mesoamerica/Central America, respectively. But the allele age obtained using the observed LD and a Bayesian approach, is relatively younger, 7,540 YBP, with a posterior probability of 99%. Although the numbers generated with these two distinct methods are compatible with an American origin of the ABCA1*230Cys allele [32], the last seems more realistic since methods based on LD, rather than frequencies, have the property of reflecting what happened to an allele more accurately [33]. Discrepancies between estimates obtained from these two approaches are usually taken as evidence that selection has increased the frequency of the allele to higher levels than expected by random genetic drift [33], [54].

#### (c.2) Detecting candidate loci for selection

**Figure 1:**
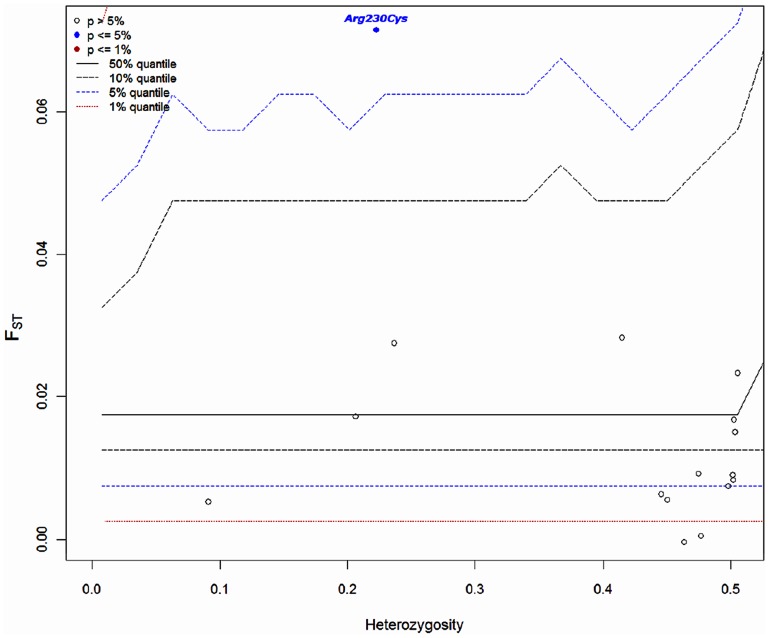
Plot of the joint *F_ST_* and *He* distributions for the Mesoamerican agriculturalist *versus* South American (agriculturalists + hunter-gatherer/foraging) groups. Each dot indicates a SNP (listed in item c.2 in the Materials and Methods section). The lines represent confidence intervals. Only the *ABCA1* locus showed significance at the 5% level (filled blue circle). Five selected SNPs were not plotted in the figure because of monomorphic sites in all subdivisions, missing data, and/or dot superposition.

Patterns of genetic diversity between populations can be used to detect loci under selection [30]. The joint distributions of *He* and *F_ST_* of the ABCA1*230 locus and 20 other SNPs (listed in item c.2 in the Materials and Methods section) were examined to test whether the ABCA1*230 locus and these SNPs departed from neutral expectation. The values obtained indicated that only the ABCA1*230 polymorphism departed significantly from neutral expectation (*p* = 0.02; Figure 1). However, when the comparisons excluded the Mesoamericans (e.g., Andean agriculturalists *vs.* South American hunter-gatherer/forager subdivisions), no significant departure from the expected under neutrality was found (data not shown). These results suggest that the ABCA1*230 allele frequencies in Mesoamerica are incompatible with a simple neutral model.

Our neutral demographic simulation analysis showed results in the same direction. The region containing the ABCA1*230 polymorphism and 19 flanking SNPs presented a slightly lower average heterozygosity than the putative neutral region dataset (0.32 vs. 0.34 respectively); but global and pairwise *F_ST_* were higher for the *ABCA1* region (global *F_ST_* 0.03 vs. 0.01; average pairwise *F_ST_* 0.05 vs. 0.01). Considering that both genomic regions were studied using the same quantity of markers and the same sampling strategy, in populations that were subjected to the same demographic history, the observed differences may occur due to diverse factors, one of them being natural selection. To test this hypothesis each dataset, summarized by the above-mentioned statistics, was also compared to each of 100,000 neutral simulations by means of Euclidian distances. The empirical dataset containing the ABCA1*230 polymorphism presented a poorer fit to neutrality than the putative neutral region dataset, showing Euclidian distances that were twofold higher than those of the neutral simulations (70.64 *vs.* 35.12). Interestingly, when the Mesoamerican agriculturalist subdivision was excluded from the analysis, this difference dramatically decreased (45.80 *vs.* 35.12). Thus, the poor fit to neutrality observed at the ABCA1*230 site and its flanking regions may be associated with the genetic pattern found in Mesoamerica.

#### (d) Allele frequencies vs. maize domestication

**Figure 2:**
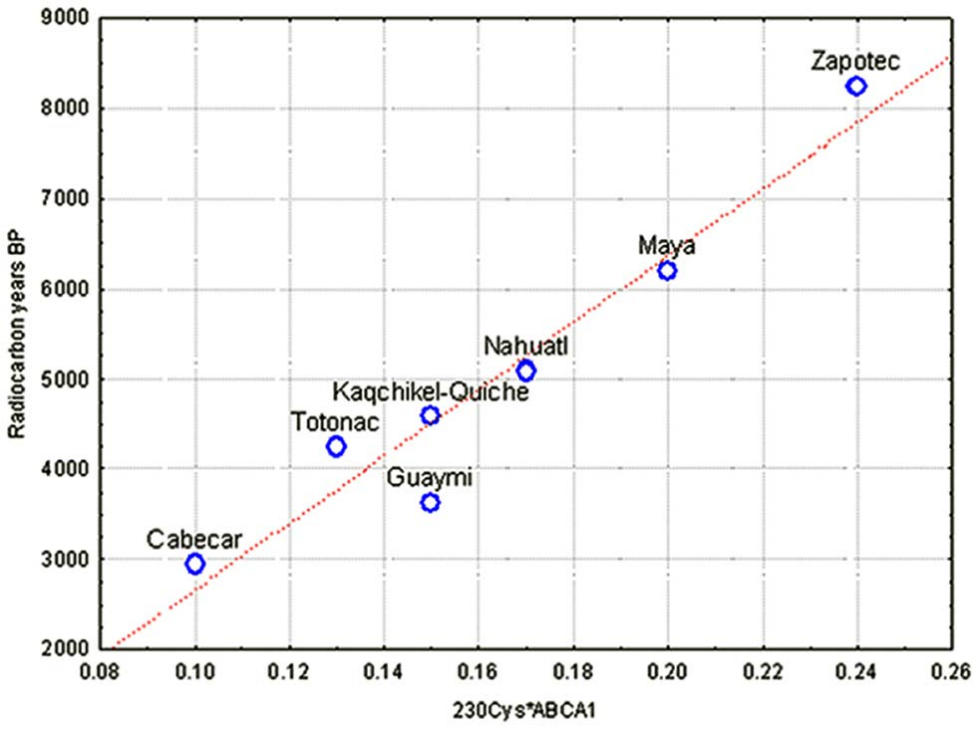
ABCA1*230Cys frequencies versus radiocarbon ages of maize domestication (*Zea* pollen relics; Blake [**42**]). Spearman’s rho value  = 0.936975 and *p* = 0.0019.

A significant correlation was observed between the ABCA1*230Cys allele frequencies and the distribution of the *Zea* pollen relics in Mesoamerica (Figure 2; r = 0.9, *p* = 0.002). It is important to note that the populations used in the correlation analysis performed here (Zapotec, Maya, Nahuatl, Kaqchikel-Quiche, Totonac, Guaymí, and Cabecar) were investigated for microsatellites and other genetic markers in previous studies conducted by our and other groups [27], [55], [56]. These studies indicated that these populations have a substantial Amerindian substrate, a generally small European contribution, and almost no African influence. For instance, Wang et al. [27] showed that the Maya and Guaymí showed the highest and the lowest numbers of individuals with some level of recent European and African admixture, respectively. This indicates an opposite trend from what would be expected if the level of admixture with non-Indians was influencing our findings, since the variant allele is absent in Europeans and Africans.

## Discussion

We can now examine some hypotheses in an attempt to explain the results and to draw the evolutionary scenario associated with the pattern of diversity of the ABCA1* Arg230Cys polymorphism.

Maize is considered the most important native crop of the Americas [44]–[53]. Several lines of evidence indicate that the Mesoamerican village lifestyle began with maize domestication [44], [45], [49]–[51], [57], [58]. Originating in the Mexican southwestern lowlands, maize journeyed southwards, traveling hand-in-hand with pottery and bringing sedentary life to the Andes, although the date of its entry, as well as the dispersion pattern of this crop into and throughout South America, remain controversial [52].

Other crops were also present in the pre-Columbian Mesoamerican civilizations (squash and beans; [50], [59]), but maize was the dietary base for most of these civilizations. For example, Benedict and Steggerda [60] showed that 75% of the calories consumed by the Mayas were derived from maize. In addition, Mesoamerica was the only region in the world where an ancient civilization lacked a domesticated herbivore. Therefore, protein from domesticated animal sources would have been scarce in Pre-Hispanic Mesoamerica in comparison to other parts of the ancient urbanized world, including the Andes [61]. As a whole, these studies demonstrated that the diet of the first Mesoamerican sedentary communities was extremely dependent on maize. These early farmers, however, suffered periods of plantation loss, questioning the common assumption that farming and sedentary lifestyle brought increased dietary stability and health homeostasis [62]. Several studies have revealed that homeostasis should have declined with sedentary farming, and bioarcheologists and paleopathologists have also detected a deterioration in Mesoamerican health indices from ∼8,000 to ∼500 years before present-YBP ([63] and references therein). Domestic crops are more vulnerable than wild ones, crowding promotes crop diseases, and storage systems often fail (estimates suggest that as much as 30% of stored food is lost even in a modern sophisticated system [62]). In other study, based on the molecular analysis of dietary diversity for three archaic Native Americans, Poinar et al. [64] found evidence that, as compared to individuals dependent on agriculture, the diet of hunter-gatherers seems to have been more varied and nutritionally sound. Clearly, a diet based on one or only a few crops should have been deleterious to health in the pre-Columbian era [65]. These different lines of evidence illustrate that the incipient farming niches of Mesoamerica, when communities of hunters/gatherers/foragers started to cultivate and domesticate wild plants, could have been remarkably unstable like those of other pre-industrial societies [6].

Based on what was discussed above, as well as in our results (allele age and neutrality/selection tests), it is reasonable to suppose that ABCA1*230Cys has an American origin and it could have had a selective advantage during the periods of food scarcity experienced by Mesoamericans during the implementation of the sedentary life style based on maize. The strong correlation between maize culture propagation and *230Cys* frequencies in this region reinforces this suggestion, even when considering that the advantage of the allele may have been lost after technological innovations had been implemented and agricultural production stabilized. Peng *et al.* [66] presented evidence for a similar case of gene-culture coevolution, suggesting that positive selection for the ADH1B*47His allele was caused by the emergence and expansion of rice domestication in East Asia.

Noteworthy is that other environmental factors may also have been involved in the distribution of the *ABCA1* alleles, since cholesterol plays an important role in various infectious processes, such as the entry and replication of Dengue virus type 2 and flaviviral infection [67]. Additionally, the ABCA1 transporter participates in infectious and/or thrombotic disorders involving vesiculation, since homozygous *ABCA1* gene deletions confer complete resistance against cerebral malaria in mice [68], [69]. These findings can be considered as additional causal factors to the ABCA1*230Cys selective sweep associated with agricultural development. A sedentary village lifestyle with a corresponding growth in the density of the local population can promote an increase in the mortality rate, particularly in children under 5 years of age [70]. For example, archeological and paleoecological evidence in Europe showed that during the Neolithic demographic transition, the causes of increased infant mortality would have included a lack of drinking water supplies, contamination by feces, emergence of highly virulent zoonoses, as well as an increase in the prevalences of other germs such as *Rotavirus* and *Coronavirus* (causing diarrhea, one of the main killers of children under 5 years of age), *Streptococcus, Staphylococcus*, *Plasmodium* (*P. falciparum* and *P. vivax*, which are believed to have emerged more recently), and *Herpesvirus* [70]. However, the real impact of the ABCA1*230Cys variant in these infectious processes will require additional functional studies.

In agreement with this historical scenario, the genetic variation in *Arg230Cys* presented a worse fit to neutrality than loci known to be neutral, indicating that selective mechanisms are necessary to explain the genetic diversity of *Arg230Cys*, especially when the Mesoamerican agriculturalist subdivision is considered in the analysis. This result also supports our hypothesis that maize domestication in Mesoamerica lead to changes in the gene pool of the natives from that region.

South America presents much more diversity in relation to habitats, people, and culture than Mesoamerica. For instance, maize arrived in South America, but apparently the level of consumption seen in Mesoamerica/Central America was rarely found there. Archaeological data indicate that only during the implantation and expansion of the Inca Empire (800–500 YBP) was the level of maize consumption important, but the level of consumption was not comparable to that of Mesoamerica/Central America [71]–[73]. Additionally, South Amerindian hunter-gatherers/foragers present lower intrapopulation genetic variation and higher levels of population structure when compared to those seen in Andean populations [27], [74]. This same tendency was also observed in the present study. These results indicate low levels of gene flow between villages/populations and low effective population sizes, favoring the role of genetic drift. Conversely, the Andean groups show opposing characteristics. These findings correlate well with distinguishing patterns of gene flow and historical effective sizes in these indigenous populations, with cultural differences, as well as with paleoclimatic and environment changes in their habitats [74]. Therefore, the significant role of random processes and/or more heterogeneous cultural and ecological scenarios makes it difficult to define a particular pattern associated with the *Arg230Cys* polymorphism in South American groups, a situation different from that in Mesoamerica.

In conclusion, our analyses demonstrate for the first time a robust correlation between a constructed niche and a selected Native American autochthonous allele. The *230Cys* allele, with a probable origin in America continent, seems have been the target for an ongoing directional selective sweep as a result of the origin and spread of the maize culture in ancient Mesoamerica.

## Supporting Information

Figure S1 **Twenty SNPs selected based on their location (chromosome 9: from position 85252250 to 85317359) inside the putative neutral region, defined by Schroeder and colleagues** [**39**] **, which comprises ∼76,000 bp around the D9S1120 locus.**
(JPG)

Table S1 **Populations included in the selection analyses.**
(DOC)

Table S2 **Allele frequency by region of 20 SNPs located around the D9S1120 locus.**
(DOCX)

Table S3 Allele frequency by region of 19 SNPs located around the ABCA1*230 locus.
(DOCX)
