# Correction: Evolutionary Responses to a Constructed Niche: Ancient Mesoamericans as a Model of Gene-Culture Coevolution

**DOI:** 10.1371/annotation/c648ffaa-47da-45e4-9cde-ef2f4c95ce29

**Published:** 2012-07-10

**Authors:** Tábita Hünemeier, Carlos Eduardo Guerra Amorim, Soledad Azevedo, Veronica Contini, Víctor Acuña-Alonzo, Francisco Rothhammer, Jean-Michel Dugoujon, Stephane Mazières, Ramiro Barrantes, María Teresa Villarreal-Molina, Vanessa Rodrigues Paixão-Côrtes, Francisco M. Salzano, Samuel Canizales-Quinteros, Andres Ruiz-Linares, Maria Cátira Bortolini

In Table 1, the heading signifying information for Andean Agriculturalist information was missing. Please see the corrected table here: 

**Figure:**
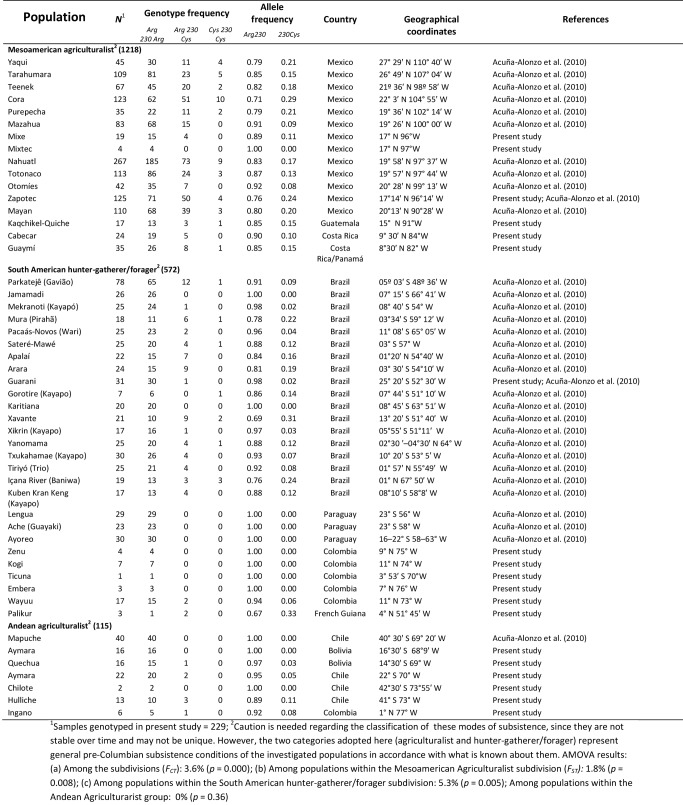


[^] 

