# Supplementary figures and images for: Evolutionary Responses to a Constructed Niche: Ancient Mesoamericans as a Model of Gene-Culture Coevolution
Source: PLoS One. 2012 Jun 21;7(6):e38862. doi: 10.1371/journal.pone.0038862 (PMC3380856; doi:10.1371/journal.pone.0038862)

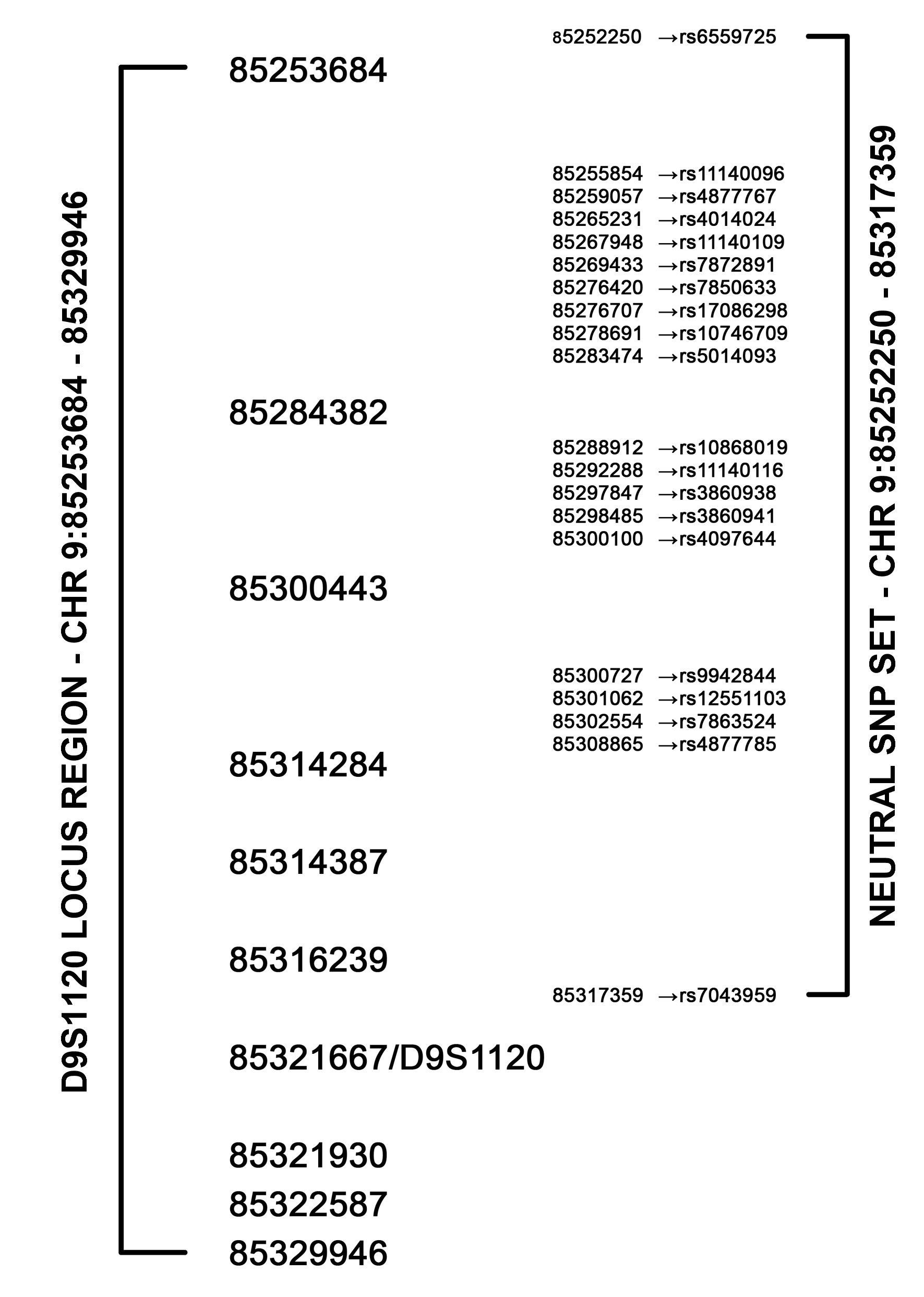

Supplement: Figure S1 — Twenty SNPs selected based on their location (chromosome 9: from position 85252250 to 85317359) inside the putative neutral region, defined by Schroeder and colleagues [39] , which comprises ∼76,000 bp around the D9S1120 locus. (JPG) [file pone.0038862.s001.jpg]
