# Supplementary material for: Evolutionary Responses to a Constructed Niche: Ancient Mesoamericans as a Model of Gene-Culture Coevolution
Source: PLoS One. 2012 Jun 21;7(6):e38862. doi: 10.1371/journal.pone.0038862 (PMC3380856; doi:10.1371/journal.pone.0038862)
Supplement: Table S1 — Populations included in the selection analyses. (DOC) [file pone.0038862.s002.doc]

| | Supplementary table 1**.** Populations included in the selection analyses | | | --- | --- | | **Population** | N | | **Mesoamerican-agriculturalist** | **68** | | Mixe | 18 | | Mixtec | 4 | | Zapotec | 14 | | Kaqchikel-Quiche | 12 | | Cabecar | 15 | | Guaymí | 5 | | **South American hunter-gatherer/forager** | **23** | | Guarani | 1 | | Zenu | 4 | | Kogi | 4 | | Ticuna | 1 | | Embera | 3 | | Wayuu | 7 | | Palikur | 3 | | **Andean agriculturalist** | **35** | | Aymara (Bolivia) | 16 | | Aymara (Chile) | 6 | | Quechua | 13 | |  |  | | **Total** | **126** | |
| --- | --- | --- | --- | --- | --- | --- | --- | --- | --- | --- | --- | --- | --- | --- | --- | --- | --- | --- | --- | --- | --- | --- | --- | --- | --- | --- | --- | --- | --- | --- | --- | --- | --- | --- | --- | --- | --- | --- | --- | --- | --- | --- | --- | --- | --- | --- |
