# Supplementary material for: Evolutionary Responses to a Constructed Niche: Ancient Mesoamericans as a Model of Gene-Culture Coevolution
Source: PLoS One. 2012 Jun 21;7(6):e38862. doi: 10.1371/journal.pone.0038862 (PMC3380856; doi:10.1371/journal.pone.0038862)
Supplement: Table S2 — Allele frequency by region of 20 SNPs located around the D9S1120 locus. (DOCX) [file pone.0038862.s003.docx]

Table S2. Allele frequency by region of 20 SNPs located around the *D9S1120* locus

|  |  | Allele Frequency | | |
| --- | --- | --- | --- | --- |
| SNP | Alleles | South America  n=23 | Mesoamerica  n=68 | Andes  N=35 |
| rs6559725 | A | 0.6875 | 0.67647 | 0.58333 |
|  | C | 0.3125 | 0.32353 | 0.41667 |
| rs11140096 | A | 0.56522 | 0.57353 | 0.44286 |
|  | G | 0.43478 | 0.42647 | 0.55714 |
| rs4877767 | C | 0.73913 | 0.76471 | 0.6 |
|  | T | 0.26087 | 0.23529 | 0.4 |
| rs4014024 | A | 0.3913 | 0.38235 | 0.41429 |
|  | G | 0.6087 | 0.61765 | 0.58571 |
| rs11140109 | C | 0.021739 | 0.16176 | 0.15714 |
|  | T | 0.97826 | 0.83824 | 0.84286 |
| rs7872891 | C | 0.65217 | 0.66912 | 0.57143 |
|  | T | 0.34783 | 0.33088 | 0.42857 |
| rs7850633 | C | 0.67391 | 0.50735 | 0.45714 |
|  | T | 0.32609 | 0.49265 | 0.54286 |
| rs17086298 | T | 1 | 1 | 1 |
| rs10746709 | C | 0.58696 | 0.45588 | 0.38571 |
|  | T | 0.41304 | 0.54412 | 0.61429 |
| rs5014093 | A | 0.30435 | 0.33088 | 0.38571 |
|  | G | 0.69565 | 0.66912 | 0.61429 |
| rs10868019 | C | 0.065217 | 0.051471 | 0.028571 |
|  | T | 0.93478 | 0.94853 | 0.97143 |
| rs11140116 | C | 1 | 1 | 1 |
| rs3860938 | C | 1 | 1 | 1 |
| rs3860941 | C | 0.6087 | 0.44853 | 0.42857 |
|  | T | 0.3913 | 0.55147 | 0.57143 |
| rs4097644 | C | 0.58696 | 0.44853 | 0.42857 |
|  | T | 0.41304 | 0.55147 | 0.57143 |
| rs9942844 | C | 0.93478 | 0.94853 | 0.97143 |
|  | T | 0.065217 | 0.051471 | 0.028571 |
| rs12551103 | C | 0.80435 | 0.91912 | 0.87143 |
|  | T | 0.19565 | 0.080882 | 0.12857 |
| rs7863524 | G | 0.56522 | 0.42647 | 0.41429 |
|  | T | 0.43478 | 0.57353 | 0.58571 |
| rs4877785 | G | 0.43478 | 0.39706 | 0.32857 |
|  | T | 0.56522 | 0.60294 | 0.67143 |
| rs7043959 | A | 0.36957 | 0.34559 | 0.28571 |
|  | G | 0.63043 | 0.65441 | 0.71429 |
