# Supplementary material for: Evolutionary Responses to a Constructed Niche: Ancient Mesoamericans as a Model of Gene-Culture Coevolution
Source: PLoS One. 2012 Jun 21;7(6):e38862. doi: 10.1371/journal.pone.0038862 (PMC3380856; doi:10.1371/journal.pone.0038862)
Supplement: Table S3 — Allele frequency by region of 19 SNPs located around the ABCA1*230 locus. (DOCX) [file pone.0038862.s004.docx]

| Table S3. Allele frequency by region of 19 SNPs located around the *ABCA1*230* locus | | | | |
| --- | --- | --- | --- | --- |
|  |  | Allele Frequency | | |
| SNP | Allele | South America  n=23 | Mesoamerica  n=68 | Andes  n=35 |
| rs2065412 | C | 0.34783 | 0.55147 | 0.44286 |
|  | T | 0.65217 | 0.44853 | 0.55714 |
| rs2515601 | C | 0.3913 | 0.24265 | 0.2 |
|  | T | 0.6087 | 0.75735 | 0.8 |
| rs2472386 | C | 0.3913 | 0.61765 | 0.45714 |
|  | T | 0.6087 | 0.38235 | 0.54286 |
| rs2274873 | G | 1 | 1 | 1 |
| rs2487054 | A | 0.3913 | 0.625 | 0.45714 |
|  | C | 0.6087 | 0.375 | 0.54286 |
| rs4149290 | A | 1 | 1 | 1 |
| rs2487039 | C | 0.52174 | 0.63235 | 0.74286 |
|  | T | 0.47826 | 0.36765 | 0.25714 |
| rs2472384 | A | 0.52174 | 0.63235 | 0.74286 |
|  | G | 0.47826 | 0.36765 | 0.25714 |
| rs2253174 | A | 0.47826 | 0.36765 | 0.25714 |
|  | G | 0.52174 | 0.63235 | 0.74286 |
| rs2230806 | A | 0.47826 | 0.36029 | 0.25714 |
|  | G | 0.52174 | 0.63971 | 0.74286 |
| rs2230805 | A | 0.5 | 0.38235 | 0.25714 |
|  | G | 0.5 | 0.61765 | 0.74286 |
| rs2249891 | A | 0.41304 | 0.61765 | 0.74286 |
|  | G | 0.58696 | 0.38235 | 0.25714 |
| rs4149281 | A | 0.54348 | 0.375 | 0.2 |
|  | G | 0.45652 | 0.625 | 0.8 |
| rs4743764 | C | 0.15217 | 0.03676 | 0.25714 |
|  | T | 0.84783 | 0.96324 | 0.74286 |
| rs1929841 | G | 0.15217 | 0.02941 | 0.17143 |
|  | T | 0.84783 | 0.97059 | 0.82857 |
| rs2000069 | C | 0.80435 | 0.84559 | 0.8 |
|  | T | 0.19565 | 0.15441 | 0.2 |
| rs2275542 | C | 0.82609 | 0.95588 | 0.8 |
|  | T | 0.17391 | 0.04412 | 0.2 |
| rs3904998 | A | 0.82609 | 0.95588 | 0.8 |
|  | G | 0.17391 | 0.04412 | 0.2 |
| rs4149268 | A | 0.17391 | 0.04412 | 0.2 |
|  | G | 0.82609 | 0.95588 | 0.8 |
